# A Long-Term Survivor of Metastatic Pancreatic Adenocarcinoma: Free of Recurrence 12 Years After Treatment of Oligometastatic Disease

**DOI:** 10.7759/cureus.1007

**Published:** 2017-02-02

**Authors:** John M Stahl, Zenta Walther, Bryan W Chang, Howard S Hochster, Kimberly L Johung

**Affiliations:** 1 Department of Therapeutic Radiology, Yale University School of Medicine; 2 Department of Pathology, Yale University School of Medicine; 3 Radiation Oncology, Torrance Memorial Medical Center; 4 Department of Medical Oncology, Yale University School of Medicine

**Keywords:** long-term survivor, metastatectomy, pancreatic adenocarcinoma, oligometastatic cancer

## Abstract

Aggressive local therapy for patients with oligometastatic pancreatic ductal adenocarcinoma (PDAC) has traditionally not been pursued due to high rates of distant progression. We describe a 62-year-old male initially presenting with resectable PDAC who underwent the Whipple procedure but developed multiple liver metastases within two months of starting adjuvant gemcitabine. Oxaliplatin was added to the regimen and complete resolution of the liver lesions resulted. He remained disease-free for five years until re-staging revealed a small lung nodule. This was resected and confirmed to be metastatic PDAC. After additional adjuvant gemcitabine, the patient remained free of recurrence for 12 years after diagnosis of metastatic disease and ultimately passed away from complications of ascending cholangitis associated with stricture at the biliary-enteric anastomosis site. He had no evidence of disease recurrence at the time of death. Next-generation sequencing of the tumor was unrevealing, showing only an activating mutation of KRAS and a deleterious mutation of tumor protein p53 (TP53). Our case suggests that while the prognosis for metastatic PDAC is poor, the population is nonetheless heterogeneous. Prognostic biomarkers are needed for the identification of patients for whom aggressive local treatment of oligometastatic PDAC may be warranted.

## Introduction

With a five year overall survival of 7.7% and median survival of 12.7 months, Stage IIB pancreatic ductal adenocarcinoma (PDAC) patients at diagnosis have a very limited prognosis [1]. Metastatic PDAC is thought to be incurable with five-year survival < 1%. Aggressive local therapy for oligometastatic PDAC has traditionally not been pursued due to the high rates of distant progression in this population. Informed consent was obtained from the patient for this study.

## Case presentation

A 62-year-old male nonsmoker presented to the emergency department with three months of persistent nausea, anorexia, jaundice, dark urine, and generalized pruritus with associated six-pound weight loss. Laboratory testing was consistent with obstructive jaundice, while abdominal ultrasound and computed tomography (CT) scan of the abdomen/pelvis revealed intra- and extrahepatic biliary ductal dilatation, a prominent pancreatic duct, and a suspicious peripancreatic lymph node without obvious mass in the pancreas.

**Figure 1:**
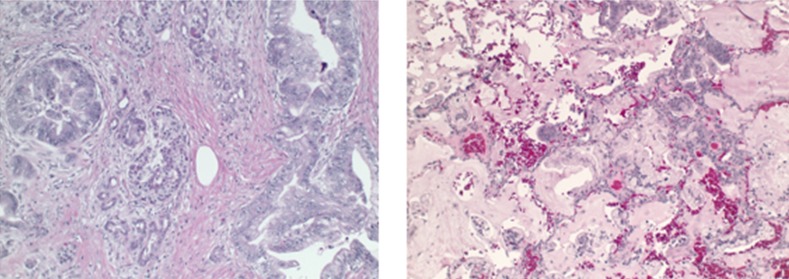
Histological appearance of the primary tumor and metastatic focus H&E slides showing the primary tumor on resection (left) and pulmonary metastases (right). The mucinous adenocarcinoma in the lung was found to be TTF-1 negative, CK7 and CK20 positive which is consistent with a pancreatic primary

Endoscopic ultrasound showed a 2.7 x 2.6 cm mass in the head of the pancreas, and fine needle aspiration was positive for adenocarcinoma. Staging showed resectable disease and no evidence of metastasis. Preoperative CA 19-9 was 81.1 U/mL (reference range: 0.0 – 37.0 U/mL) and CEA was 3.1 ng/mL (reference range: 0.0 – 3.0 ng/mL). He underwent diagnostic laparoscopy with pylorus-preserving Whipple procedure. Pathology revealed a moderately differentiated adenocarcinoma measuring 3.7 cm and invading into the pancreatic parenchyma and through the muscularis propria of the duodenum into the submucosa (Figure 1). There was lymphovascular and extensive perineural invasion identified, margins were negative, and five out of 19 lymph nodes were involved with adenocarcinoma. The tumor was staged IIB (pT3 N1 M0). Adjuvant gemcitabine was started one month postoperatively at 1,200 mg/m^2^ weekly for three of four weeks.

**Figure 2:**
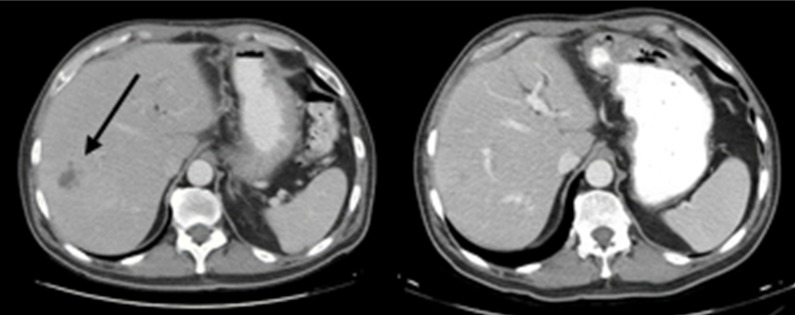
Axial CT images showing response of presumed liver metastases to chemotherapy The largest low attenuation liver lesion at the time of diagnosis (left with arrow), and repeat imaging 3.5 months later after receiving gemcitabine and oxaliplatin (right) showing interval resolution

After completing his first three weeks of chemotherapy, he was admitted with neutropenic fever and found to have developed liver lesions on CT abdomen. Three low attenuating masses were identified, the largest 3.1 cm in greatest dimension with subtle rim enhancement concerning for possible liver metastases (Figure 2). The biopsy was not pursued but oxaliplatin was added to the palliative chemotherapy regimen at 100 mg/m^2^, while gemcitabine was continued at 1,000 mg/m^2^ every two weeks. Restaging CT scan 3.5 months after diagnosis of metastases noted a complete resolution of the liver lesions. He completed 22 infusions (over the course of about one year) of this therapy and was offered a treatment holiday thereafter when positron emission tomography (PET) scan showed no evidence of disease.

**Figure 3:**
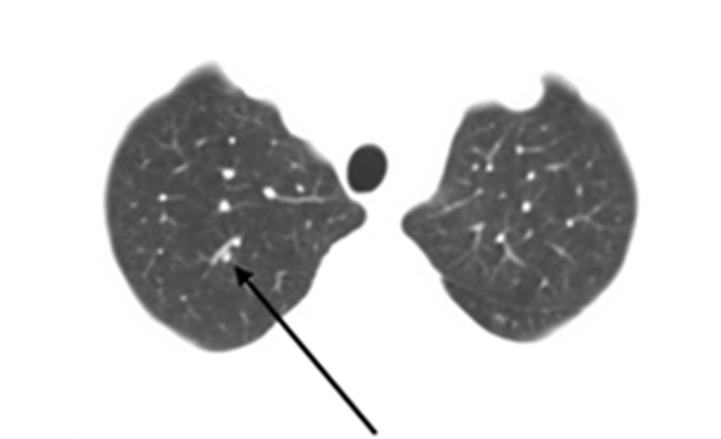
Axial CT image showing metastatic pulmonary nodule Right upper lobe lung nodule found to be metastatic pancreatic adenocarcinoma five years after initial presentation. The black arrow points to the lesion adjacent to a blood vessel.

He remained free of disease and off systemic therapy for five years at which point a re-staging CT chest showed a 6 mm nodule in the upper lobe of the right lung, which was felt to be isolated metastatic disease (Figure 3). Radiotherapy was not considered as a local management approach due to the need for tissue diagnosis. Cardiothoracic surgery performed a video-assisted thoracoscopic right upper lobectomy when the nodule could not be located intraoperatively with pathology showing a well-differentiated mucinous adenocarcinoma (1 cm in size). Staining was consistent with pancreatic primary (CK7/CK20 positive, TTF-1, and CDX-2 negative) and margins were negative. Postoperatively, he was treated with five cycles of adjuvant gemcitabine, which was eventually discontinued due to transaminitis, with no additional therapy. He remained off treatment for six additional years, with surveillance CT scans approximately every six months showing no evidence of recurrence.

About 12 years after diagnosis of metastatic disease, the patient was admitted to the hospital and ultimately passed away from complications of ascending cholangitis associated with stricture at the biliary-enteric anastomosis site. The CT scan of the chest, abdomen, and pelvis during this hospitalization did not show evidence of disease recurrence. Biopsy of the stricture site showed no evidence of malignancy. Nucleic acids were extracted from microdissected paraffin sections of the pancreatic tumor as well as an adjacent benign lymph node. Next-generation sequencing libraries of the tumor and normal samples were constructed by the Yale Tumor Profiling Laboratory, using the Oncomine Comprehensive Assay (Life Technologies, ThermoFisher Scientific). This assay examines tumor DNA for mutations and/or amplifications in 134 cancer-related genes. The assay also examines tumor ribonucleic acid (RNA) for the presence of 183 fusion transcripts involving 22 oncogenic driver genes. Sequencing was performed using the Ion Torrent platform (ThermoFisher Scientific) and data were analyzed using Ion Torrent software as well as custom software developed by the laboratory. An activating mutation of KRAS (G12R) and a deleterious mutation of TP53 were identified in the primary pancreatic tumor. The primary tumor was also analyzed for DNA mismatch repair protein expression by immunohistochemistry and showed no abnormality; furthermore, the primary tumor showed no evidence of microsatellite instability in a direct polymerase chain reaction-based test. There was no inactivation of SMAD4. Notably, there was no evidence of a germline mutation in BRCA2 in either the tumor or the normal tissue sample. Pyrosequencing of KRAS codons 12-13 in the presumed metastatic lung tumor specimen confirmed the presence of a KRAS G12R mutation, providing strong evidence that this pulmonary lesion did indeed originate from the initial pancreatic primary tumor.

## Discussion

We describe a rare case of oligometastatic PDAC treated aggressively with systemic platinum-based chemotherapy and pulmonary metastasectomy resulting in durable survival with no evidence of disease at the time of death over 12 years after diagnosis of metastases. While there is no established role for aggressive local therapy in the setting of metastatic PDAC, the prolonged survival seen in our patient suggests that rare PDAC patients who achieve systemic disease control would benefit from local therapy for the oligometastatic disease.

First described in 1995 by Hellman and Weichselbaum, the oligometastatic state represents a stage of cancer progression when very few detectable metastases exist and the ability of malignant cells to acquire widespread metastatic potential is limited. It followed that the aggressive treatment of isolated metastatic foci could prevent future seeding of widespread metastatic disease (a challenge to the traditional belief that patients with the oligometastatic disease should only be treated with palliative intent). Improved local control and overall survival have been reported with isolated surgical metastasectomy, especially for liver and lung metastases [2-3].

Our patient's outcome after surgical resection of metastatic disease suggests that aggressive local therapy should not be excluded for some oligometastatic patients with PDAC. Only a handful of PDAC patients with notably long survival after diagnosis of metastatic disease have been described in the literature. Two patients found to have liver metastases at diagnosis survived eight and 10 years after receiving primarily systemic therapy [4-5]. A patient with a single liver and brain metastasis (both resected) survived 14 years after the first diagnosis of metastatic disease [6].

Retroactive sequencing of the initial surgical specimen for our patient showed no unusual mutation patterns. Mutation of the KRAS oncogene in codon-12 is present in the majority of PDAC. Our patient’s particular KRAS mutation, G12R, was described in 21 out of 147 (14%) of all codon-12 KRAS mutations in a group of advanced pancreatic adenocarcinoma patients [7]. It has no known association with improved prognosis. We initially suspected that our patient harbored a BRCA2 mutation, given his family history (maternal breast cancer before the age of 50) and excellent response to platinum chemotherapy. The BRCA2 gene is involved in DNA double-strand break repair via homologous recombination (HR) and patients heterozygous for this highly penetrant mutation are at increased risk for multiple cancers including PDAC. There are small retrospective studies, case reports [8], and emerging data reporting positive outcomes in BRCA mutant PDAC patients treated with platinum agents. Our patient did not harbor a BRCA2 mutation and other genes relevant to PDAC were found to be wild-type. No other genes involved in DNA repair pathways were found to be mutated. SMAD4 (functioning in the transforming growth factor beta receptor pathway) is inactivated in about half of pancreatic cancers and is associated with higher rates of distant metastases [9]. Although our patient did develop distant metastases, we did not find an SMAD4 mutation in his PDAC, perhaps portending a more favorable outcome. The RBM10 gene was not assessed by our sequencing panel, though it was recently found to be associated with improved survival when mutated in a cohort of 109 patients with resected PDAC [10]. Thus, our genomic testing revealed neither a BRCA2 germline mutation nor somatic mutations in the tumor that could have explained the patient’s unusually favorable outcome.

## Conclusions

While the prognosis for metastatic PDAC is generally quite bleak, the population is nonetheless heterogeneous. Rare patients may have curable disease with aggressive systemic therapy and resection, though we did not find a genetic alteration to explain this biology. Future investigation may identify mutations and other biomarkers of good prognosis that would help to elucidate the mechanisms that underlie the favorable outcomes of patients such as ours. Such markers could aid in the prospective identification of patients for whom aggressive local treatment of oligometastatic PDAC may be warranted.
